# Expansion of clinico-genetic spectrum of *PRDX3* disease: a literature review with two additional cases

**DOI:** 10.1093/braincomms/fcad233

**Published:** 2023-08-28

**Authors:** Jaeso Cho, Jihoon G Yoon, Seungbok Lee, Sheehyun Kim, Soo Yeon Kim, Man Jin Kim, Jangsup Moon, Jong-Hee Chae

**Affiliations:** Department of Genomic Medicine, Seoul National University Hospital, Seoul 03080, Republic of Korea; Department of Genomic Medicine, Seoul National University Hospital, Seoul 03080, Republic of Korea; Department of Genomic Medicine, Seoul National University Hospital, Seoul 03080, Republic of Korea; Department of Genomic Medicine, Seoul National University Hospital, Seoul 03080, Republic of Korea; Department of Genomic Medicine, Seoul National University Hospital, Seoul 03080, Republic of Korea; Department of Pediatrics, Seoul National University College of Medicine, Seoul03080, Republic of Korea; Department of Genomic Medicine, Seoul National University Hospital, Seoul 03080, Republic of Korea; Department of Laboratory Medicine, Seoul National University Hospital, Seoul 03080, Republic of Korea; Department of Genomic Medicine, Seoul National University Hospital, Seoul 03080, Republic of Korea; Department of Neurology, Seoul National University Hospital, Seoul 03080, Republic of Korea; Department of Genomic Medicine, Seoul National University Hospital, Seoul 03080, Republic of Korea; Department of Pediatrics, Seoul National University College of Medicine, Seoul03080, Republic of Korea

## Abstract

**Graphical Abstract:**
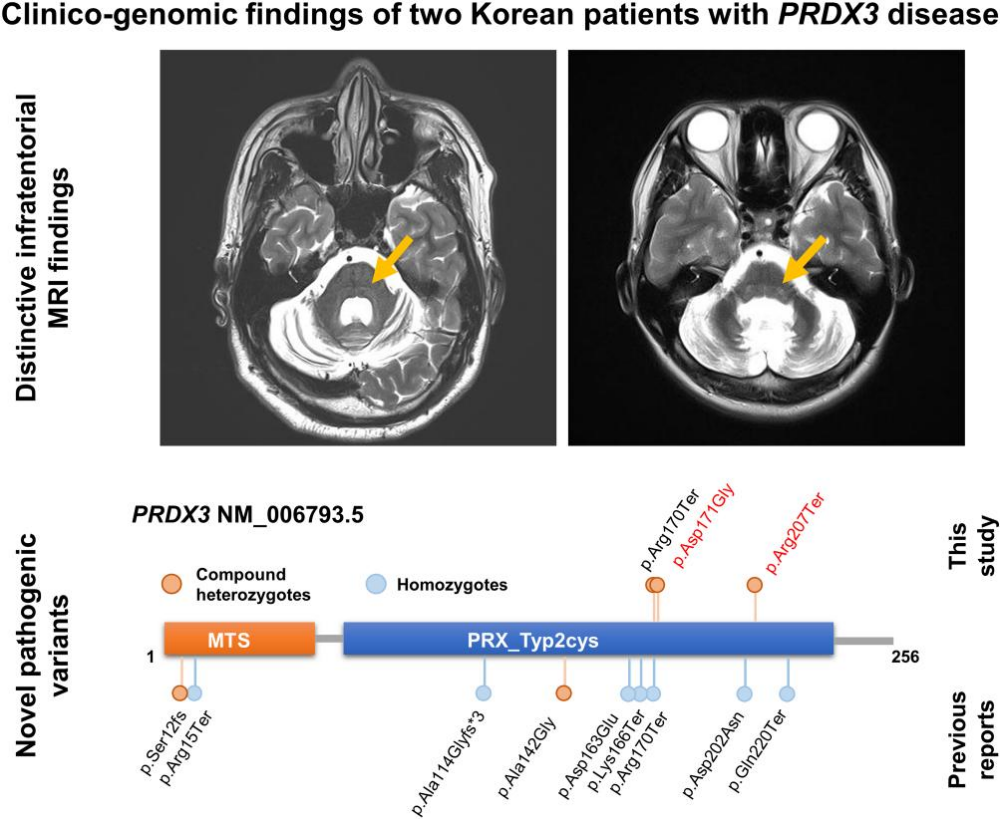

## Teaser

Cho *et al*. identified two Korean ataxia patients with novel variants, thereby broadening the clinico-genomic findings of *PRDX3* disease. The novel variants (Asp171Gly and Arg207Ter) were found in compound heterozygotes with the previously reported variant (Arg170Ter). Identification of these pathogenic *PRDX3* variants in East Asians highlights the need for increased awareness of *PRDX3* disease.

## Introduction

Peroxiredoxin III (*PRDX3*) is a crucial antioxidant protein that plays a vital role in the mitochondria by detoxifying reactive oxygen species. Recently, a study shed light on the bi-allelic variations found in the *PRDX3* gene that causes spinocerebellar ataxia, autosomal recessive type 32 [SCAR32; Online Mendelian Inheritance in Man (OMIM) #619862], often accompanied by characteristic MRI signal changes in the infratentorial region.^1,2^ To date, *PRDX3* disease has been reported in nine cases from Arabian, Indian and European populations.^2–4^ In this report, we aim to further expand the genomic and clinical spectrum of *PRDX3* disease by presenting two unrelated Korean patients with cerebellar ataxia. These patients exhibited both novel compound heterozygous variants and distinct infratentorial MRI signal changes, further contributing to the growing body of knowledge on this rare disease.

## Materials and methods

Whole-exome sequencing (WES) was performed for 2 unrelated probands including 1 trio and 1 proband-only case from 154 undiagnosed ataxia patient cohorts. The detailed method for data generation and processing was described elsewhere.^5^ Briefly, generated reads were aligned to the human reference genome hg38 and processed according to the best practice of Genome Analysis Toolkit.^6^ The ANNOVAR program was used to annotate genomic information^7^; population allele frequencies were obtained from the Genome Aggregation Database (gnomAD)^8^ and Korean Variant Archive 2 (KOVA2).^9^ Variants were interpreted based on the American College of Medical Genetics and Genomics/Association for Molecular Pathology (ACMG/AMP) criteria. Informed consent was obtained from the participants according to the Declaration of Helsinki, and the study was approved by the Institutional Review Board of Seoul National University Hospital (IRB No.1406-081-588).

## Results

### Clinical presentations and MRI findings

**Figure 1:**
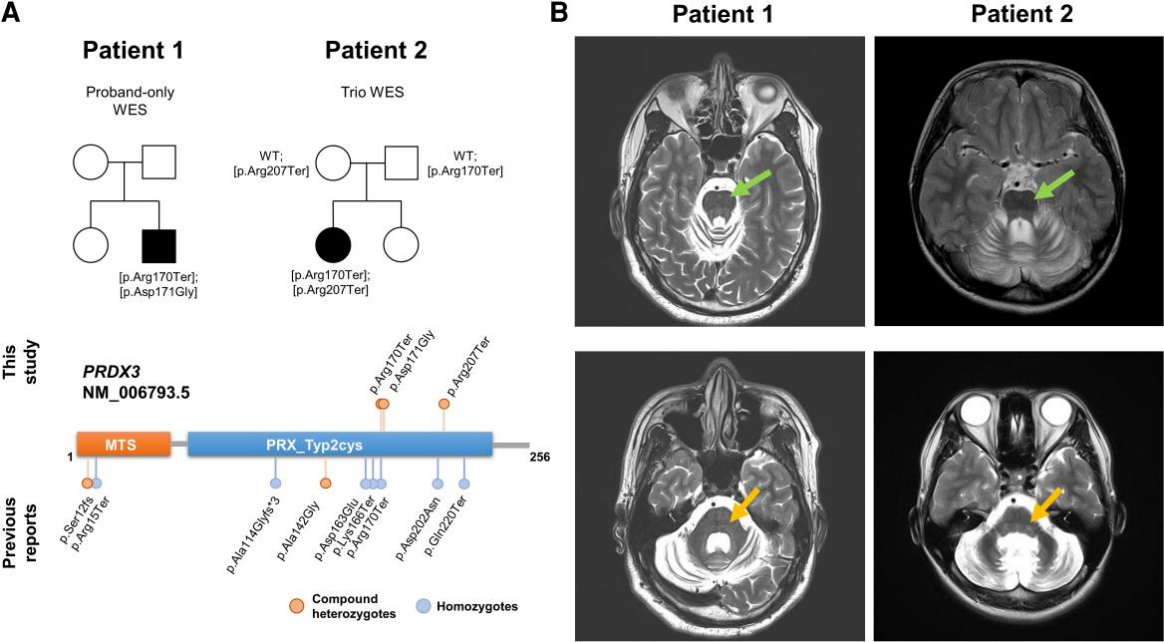
**Radio-genomic findings of ataxia patients with *PRDX3* disease identified in this study.** (**A**) Pedigree and variant information of our patients. (**B**) Brain MRI findings of Patients 1 and 2. Transverse stripes of the pons are marked with arrows in both patients (upper panel). T_2_ hyperintensity of the reticular formation and dentate nucleus are marked with arrows in both patients (lower panel).

Patient 1 is a 46-year-old Korean male patient. His older sister had no neurological symptoms and reported no other familial history (Fig. 1A). He did not exhibit any signs of motor or cognitive developmental delay during his childhood. At the age of 25, he began to experience both hand tremors and gait ataxia, which later progressed to dysarthria at the age of 35. At age 45, the patient visited our clinic due to a worsening of hand tremors, gait ataxia and dysarthria. During the neurological examination, Babinski’s sign was present but deep tendon reflexes were within the normal range. Routine cerebellar function tests showed normal results, but bilateral swaying gait was observed. No abnormal laboratory findings were observed, including in the cerebrospinal fluid (CSF) exam. The patient did not exhibit hypokinetic/hyperkinetic movements or any sensory/cognitive/hearing impairments during the last follow-up at the age of 46. The Scale for the Assessment and Rating of Ataxia (SARA) score was 6 at age 46, as shown in Table 1. In the MRI taken at age 45, T2 hyperintensity in reticular formation and dentate nucleus as well as transverse stripes of the pons were observed (Fig. 1B).

**Table 1:** Clinical characteristics of *PRDX3* disease patients

	Previously reported patients (*n* = 9)^2–4^	This study	
		Patient 1	Patient 2
Age at onset (median in years)	Infantile—35 years (15)	25	11
Gender	6 males, 3 females	Male	Female
Ethnicity	5 Arabians, 3 Europeans, 1 Indian	Korean	Korean
Consanguinity	6 (consanguine family), 1 (nonconsanguine family), 2 unknowns	No	No
Inheritance	8/9 homozygotes, 1/9 compound heterozygotes	Compound heterozygotes	Compound heterozygotes
Delayed motor milestones	3/9	No	No
Gait ataxia onset range (median in years)	9/9, 19 months—35 years (21)	Yes (25)	Yes (11)
Upper limb ataxia onset range (median in years)	9/9, 19 months—36 years (22)	Yes (25)	Yes (13)
Dysarthria onset range (median in years)	8/9, 19 months—35 years (27.5)	Yes (35)	Yes (15)
Dysphagia onset range (median in years)	6/9, 25–38 years (30.5)	No	No
SARA at the last follow-up (median) [age at the last follow-up, median in years]	12–40 (13.5) [35]	6 (46)	8 (24)
Oculomotor signs	9/9	No	No
Cerebellar dysarthria	8/9	Yes	Yes
Hypokinetic features	3/9	No	No
Hyperkinetic features	4/9	No	No
Pyramidal signs	1/9	Yes	No
Muscle weakness	2/9	No	No
Sensory impairment	1/9	No	No
Cognitive impairment	1/9	No	Yes
Hearing impairment	1/9	No	No
MRI findings			
Volume reduction of superior cerebellar peduncle	9/9	Yes	Yes
T_2_ hyperintensity in formation reticularis	2/9	Yes	Yes
T_2_ hyperintensity of dentate nucleus	2/9	Yes	Yes
Transverse stripes of the pons	1/9	Yes	Yes
T_2_ hyperintensity of cortical grey matter of the cerebellum	2/9	Yes	Yes
T_1_ hyperintensity of the substantia nigra	1/9	No	No
T_2_ hyperintensity of medullary olives	2/9	No	No

Patient 2 is a 24-year-old Korean female patient. Her younger sister had no neurological symptoms and reported no other family history (Fig. 1A). Ataxic gait was prominently observed from the age of 11 years, followed by the onset of both hands tremors at age 13 and dysarthria at age 15. She exhibited mild cognitive impairment at the age of 13, as indicated by a full-scale intelligence quotient (FSIQ) score of <75. No definite motor delays were noted during her early development. The patient’s neurological symptoms stopped progressing at age 19 and remained stationary thereafter. At age 24, the patient had a SARA score of 8 (Table 1). The patient did not exhibit hypokinetic/hyperkinetic movements or any sensory/cognitive/hearing impairment during the last follow-up at the age of 24. Imaging studies revealed a T2 hyperintensity in the reticular formation, and dentate nucleus, as well as transverse stripes of the pons, was observed (Fig. 1B).

### Genetic findings

Targeted sequencing for known 41 ataxia genes and evaluation for spinocerebellar ataxia (SCA) short-tandem repeats including SCA1, 2, 3, 6, 7, 8 and 17, and dentatorubro-pallidolusian atrophy (DRPLA) provided negative results for both patients. To further investigate the genetic basis of their condition, we performed WES and analysed ∼7,000 Mendelian disorders reported in the OMIM database.

For Patient 1, our analysis revealed two compound heterozygous variants in the *PRDX3* gene located in trans conformation: c.508C > T, p.Arg170Ter, a known disease-causing variant found in previous studies in homozygous alleles,^1^ and a novel variant c.512A > G, p.Asp171Gly, which was located 4 bp away from the reported nonsense variant. The configuration of these two alleles could be identified without family studies, as they were phased in different reads (see Supplementary Fig. 1). Initially, the novel variant (p.Asp171Gly) was classified as a variant of uncertain significance (VUS). However, subsequent computational predictions strongly suggested a highly deleterious effect for this variant, as indicated by a Combined Annotation Dependent Depletion (CADD) score of 27.6. Furthermore, analysis of evolutionary conservation revealed that the amino acid residue at position 171 is highly conserved among various species, as denoted by a GERP++ score of 5.19. Considering these *in silico* predictions, coupled with the presence of phenotypic similarities, distinctive MRI findings and segregation analysis, we reclassified this variant as a likely pathogenic variant. For Patient 2, we conducted a trio WES, and we identified two nonsense variants in the *PRDX3* genes. Our analysis confirmed that the previously reported p.Arg170Ter variant was inherited from her father, whereas the novel p.Arg207Ter variant was inherited from her mother. Her asymptomatic younger sister was not evaluated for genetic testing. Population frequencies of all reported pathogenic variants to date based on gnomAD, KOVA2 and our in-house database of 4,880 Koreans are in Supplementary Table 1.

## Discussion

Since the initial description of *PRDX3* disease in 2021, nine patients with varying onset ages ranging from infantile onset to 55 years have been reported. All of these patients presented with gait ataxia, and most eventually developed upper limb ataxia, dysarthria and/or dysphagia. The disease appeared to progress in a caudal-to-rostral spatial pattern, as suggested by Rebelo *et al.*^1^ as well as the patients in this study. Most patients displayed oculomotor signs, with a few also exhibiting hypokinetic features, hyperkinetic features and muscle weakness. Sensory, cognitive and hearing impairments as well as pyramidal signs during neurological examination were rarely reported (Table 1). Our study reports two additional patients who displayed caudal-to-rostral spatial disease spread, with one showing mild cognitive impairment and the other exhibiting pyramidal signs. Although most patients displayed a ‘neurodegenerative’ phenotype, a single case with a ‘neurodevelopmental’ component was reported, wherein the overall disease severity trajectory improved slowly over time.^1^ Patient 2 also displayed a caudal-to-rostral spatial spread of disease; however, the overall disease severity trajectory showed improvement as no symptoms worsened after the age of 19. The inclusion of our patient with an improving phenotype provides strong evidence for expanding the phenotypic spectrum of *PRDX3* disease. Only a few reports have described the MRI features of *PRDX3* disease, including T2 signal changes in the reticular formation, dentate nucleus and transverse stripes of the pons, which are not typically seen in other hereditary degenerative ataxia. Our patients also exhibited distinctive T2 signal changes which therefore strengthen the MRI spectrum of *PRDX3* disease.

The genetic findings identified in our study indicated that the p.Arg170Ter variant is the most frequent pathogenic hit (three different cases have been identified including our two cases) in the *PRDX3* disease (Supplementary Table 1). Additionally, our study has revealed two novel variants, p.Asp171Gly and p.Arg207Ter, in the Korean population. These variants have not been previously reported, thus expanding the genetic spectrum of *PRDX3* disease. The p.Asp171Gly was found only in the East Asian population, while the p.Arg207Ter variant was also reported in Europeans in the gnomAD. Particularly, both patients presented compound heterozygotes alleles highlighting the importance of consideration for compound heterozygote modes during diagnostic evaluation in nonconsanguineous populations. Considering the allele frequencies of the 3 identified variants reported in the KOVA2, *PRDX3* disease patients are estimated to be 0.34 individuals per million (at least 17 patients in the Republic of Korea), indicating that more efforts should be made in identifying *PRDX3* disease.

Knockdown of *PRDX3* in a cellular model results in reduced levels of glutathione peroxidase activity—elucidating its key role in detoxifying pathways of H_2_O_2_. The importance of reactive oxygen species in the pathomechanism of many neurodegenerative disorders suggests the importance of its pathway. A single case of clinical improvement after treatment with idebenone in a patient with homozygous *TXN2* variants, a gene closely related to the efficient cycling of *PRDX3*, suggests a possible therapeutic option may exist in *PRDX3* disease patients and calls for increased awareness of the disease.^10^

In this study, we aimed to expand the understanding of *PRDX3* disease by investigating its phenotypic and imaging spectrum. Our findings not only contribute to a better characterization of the disease but also highlight the need for increased awareness of *PRDX3* disease in undiagnosed cerebellar ataxia patients. While the majority of cerebellar ataxia cases follows an autosomal dominant inheritance pattern, patients diagnosed with recessive *PRDX3* disease may feel relieved that the disease is not likely to be passed on to their offspring. This underscores the importance of raising awareness about the disease, as early diagnosis and treatment can potentially help affected individuals both in genetic counselling and exploring possible therapeutic options. To our best knowledge, this is the first report of *PRDX3* disease in the East Asian population. *PRDX3* disease should be strongly suspected in cerebellar ataxia patients with caudal-to-rostral spatial spread pattern and/or distinctive infratentorial MRI findings regardless of one’s ethnical background.

## Supplementary Material

fcad233_Supplementary_Data

## Data Availability

The data that support the findings of this study are available from the corresponding author, upon reasonable request.
