# Supplementary material for: Expansion of clinico-genetic spectrum of PRDX3 disease: a literature review with two additional cases
Source: Brain Commun. 2023 Aug 28;5(5):fcad233. doi: 10.1093/braincomms/fcad233 (PMC10507740; doi:10.1093/braincomms/fcad233)
Supplement: fcad233_Supplementary_Data [file fcad233_supplementary_data.docx]

**Supplementary Material**

Expansion of clinico-genetic spectrum of *PRDX3* disease: a literature review with two additional cases

Jaeso Cho^1,†^, Jihoon G. Yoon^1,†^, Seungbok Lee^1^, Sheehyun Kim^1^, Soo Yeon Kim^1,4^, Man Jin Kim^1, 2^, Jangsup Moon^1,3,*^, Jong-Hee Chae^1,4,*^

1 Department of Genomic Medicine, Seoul National University Hospital, Seoul, Republic of Korea.

2 Department of Laboratory Medicine, Seoul National University Hospital, Seoul, Republic of Korea.

3 Department of Neurology, Seoul National University Hospital, Seoul, Republic of Korea.

4 Department of Pediatrics, Seoul National University College of Medicine, Seoul, Republic of Korea.

^†^These authors contributed equally to this work

*These authors jointly directed this work

Correspondence to: Jangsup Moon, MD, PhD

Department of Genomic Medicine and Department of Neurology, Seoul National University Hospital, 101 Daehak-ro, Jongno-gu, Seoul, Republic of Korea.

E-mail: [jangsup.moon@gmail.com](mailto:jangsup.moon@gmail.com)

Correspondence may also be sent to: Jong-Hee Chae, MD, PhD

Department of Genomic Medicine and Department of Pediatrics, Seoul National University Hospital, 101 Daehak-ro, Jongno-gu, Seoul, Republic of Korea.

E-mail: [chaeped1@snu.ac.kr](mailto:chaeped1@snu.ac.kr)

**Running title**: Expansion of clinicogenetic spectrum of *PRDX3* disease


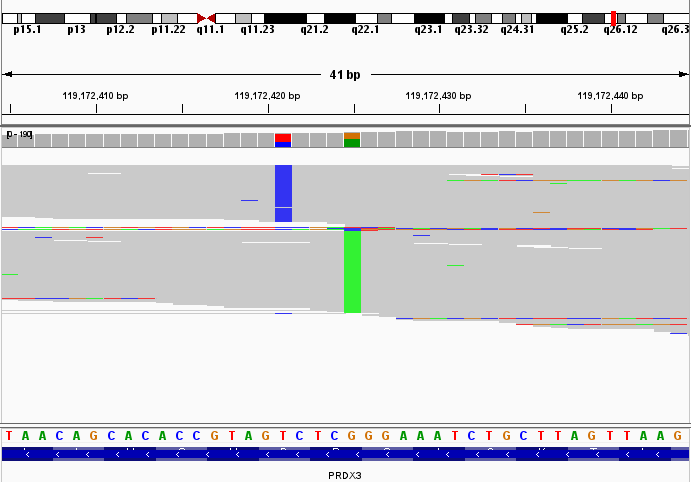


**Supplementary Figure 1. Integrative Genome Viewer findings of *PRDX3* c.508C>T (p.Arg170Ter) and c.512A>G (p.Asp171Gly) variants in Patient 1.**

The two variants were phased on two different haplotypes, which could be distinguished in short-read sequencing due to the close distance (4 base pairs).

**Supplementary Table 1. Population allele frequencies of pathogenic variants identified in 11 patients with *PRDX3* disease.**

| **CDS change** | **AA change** | **Allele counts** | | **Identified cases** | **gnomAD** | | | **KOVA2** | **In-house Database**  **(*n* = 4,880)** |
| --- | --- | --- | --- | --- | --- | --- | --- | --- | --- |
|  |  |  |  |  | **ALL** | **EUR** | **EAS** |  |  |
| c.37-2AA>G | splicing junction | 1 | 1 | | 0 | 0 | 0 | 0 | 0 |
| c.43C>T | p.Arg15Ter | 2 | 1 | | 1.99 × 10^-5^ | 2.65 × 10^-5^ | 0 | 0 | 0 |
| c.340dupG | p.Ala114Glyfs*3 | 2 | 1 | | 0 | 0 | 0 | 0 | 0 |
| c.425C>G | p.Ala142Gly | 1 | 1 | | 5.57 × 10^-5^ | 9.68 × 10^-5^ | 0 | 0 | 0 |
| c.489C>G | p.Asp163Glu | 2 | 1 | | 0 | 0 | 0 | 0 | 0 |
| c.496A>T | p.Lys166Ter | 2 | 1 | | 0 | 0 | 0 | 0 | 0 |
| c.508C>T | p.Arg170Ter | 4 | 3 | | 3.18 × 10^-5^ | 7.03 × 10^-5^ | 0 | 1.47 × 10^-4^ | 2.05 × 10^-4^ |
| c.512A>G | p.Asp171Gly | 1 | 1 | | 6.57 × 10^-6^ | 0 | 1.92 × 10^-4^ | 1.47 × 10^-4^ | 3.07 × 10^-4^ |
| c.604G>A | p.Asp202Asn | 4 | 2 | | 4.38 × 10^-5^ | 7.92 × 10^-5^ | 0 | 0 | 0 |
| c.619C>T | p.Arg207Ter | 1 | 1 | | 7.95 × 10^-6^ | 0 | 5.44 × 10^-5^ | 2.86 × 10^-4^ | 0 |
| c.658C>T | p.Gln220Ter | 2 | 1 | | 3.98 × 10^-6^ | 8.79 × 10^-6^ | 0 | 0 | 0 |

CDS: Coding Seqeuence, AA: Amino Acid: gnomAD: Genome Aggregation Database, EUR: European (non-Finnish) in gnomAD, EAS: East Asian in gnomAD, KOVA2: Korean Variant Archive
